# Mendelian Randomization Analysis of the Causal Effect of Cigarette Smoking on Hospital Costs

**DOI:** 10.1093/ntr/ntae089

**Published:** 2024-04-17

**Authors:** Padraig Dixon, Hannah Sallis, Marcus Munafò, George Davey Smith, Laura Howe

**Affiliations:** Nuffield Department of Primary Care Health Sciences, University of Oxford, UK; MRC Integrative Epidemiology Unit, University of Bristol, UK; MRC Integrative Epidemiology Unit, University of Bristol, UK; School of Psychological Science, University of Bristol, UK; MRC Integrative Epidemiology Unit, University of Bristol, UK; School of Psychological Science, University of Bristol, UK; NIHR Biomedical Research Centre, University of Bristol, UK; MRC Integrative Epidemiology Unit, University of Bristol, UK; NIHR Biomedical Research Centre, University of Bristol, UK; Population Health Sciences, University of Bristol, UK; MRC Integrative Epidemiology Unit, University of Bristol, UK; Population Health Sciences, University of Bristol, UK

## Abstract

**Introduction:**

Knowledge of the impact of smoking on health care costs is important for establishing the external effects of smoking and for evaluating policies intended to modify this behavior. Conventional analysis of this association is difficult because of omitted variable bias, reverse causality, and measurement error.

**Aims and Methods:**

We approached these challenges using a Mendelian Randomization study design; genetic variants associated with smoking behaviors were used in instrumental variables models with inpatient hospital costs (calculated from electronic health records) as the outcome. We undertook genome-wide association studies to identify genetic variants associated with smoking initiation and a composite smoking index (reflecting cumulative health impacts of smoking) on up to 300 045 individuals (mean age: 57 years at baseline, range 39–72 years) in the UK Biobank. We followed individuals up for a mean of 6 years.

**Results:**

Genetic liability to initiate smoking (ever vs. never smoking) was estimated to increase mean per-patient annual inpatient hospital costs by £477 (95% confidence interval (CI): £187 to £766). A one-unit change in genetic liability to the composite smoking index (range: 0–4.0) increased inpatient hospital costs by £204 (95% CI: £105 to £303) per unit increase in this index. There was some evidence that the composite smoking index causal models violated the instrumental variable assumptions, and all Mendelian Randomization models were estimated with considerable uncertainty. Models conditioning on risk tolerance were not robust to weak instrument bias.

**Conclusions:**

Our findings have implications for the potential cost-effectiveness of smoking interventions.

**Implications:**

We report the first Mendelian Randomization analysis of the causal effect of smoking on health care costs. Using two smoking phenotypes, we identified substantial impacts of smoking on inpatient hospital costs, although the causal models were associated with considerable uncertainty. These results could be used alongside other evidence on the impact of smoking to evaluate the cost-effectiveness of antismoking interventions and to understand the scale of externalities associated with this behavior.

## Introduction

Over one billion people smoked tobacco products—mostly cigarettes—in 2015 (1). Despite declines in prevalence in many industrialized countries,^1–3^ smoking continues to be associated with substantial morbidity and mortality.^4,5^ Smoking is arguably the most damaging of all health behaviors^6,7^ and is associated with a variety of adverse economic and socio-economic outcomes.^8^ Here we study the causal association of smoking with one such economic outcome: health care costs.

The association between smoking and health care costs is important.^7^ Accurate estimates of the health care expenditure attributable to smoking are necessary to calculate the external effects associated with smoking, and these externalities inform and underpin many government interventions intended to prevent smoking.^9–11^ Causal evidence on the effect of smoking on health care costs is also necessary for the robust evaluation of specific interventions that aim to prevent smoking and to treat its downstream consequences. Decision making by individual smokers may be improved with better information about the non-health consequences of smoking.^12^

However, establishing the causal effect of cigarette smoking on health care costs is challenging. Observed associations of smoking with health care costs may arise because smoking is indeed a cause of health care costs, because smoking is itself partly determined by health care costs, or because smoking is associated with causes or consequences of processes that influence health care costs. In general, it is not clear if the factors that may predispose an individual to smoke are themselves independent determinants of health care costs. For example, smoking tends to cluster with other behaviors known or suspected to affect health care costs, including high body mass index (BMI), poor diet, alcohol consumption, and low-physical activity.^13–17^ Smoking is also heavily socially patterned; globally, lower socio-economic status groups are more likely to smoke than higher status groups^18,19^ although this overall picture conceals variation over time and within regions. For example, in the first half of the 20th century, smoking prevalence was highest amongst higher socio-economic groups, but this pattern has now reversed.^20^ Similar patterns of greater smoking prevalence amongst high-income groups are observed in some low-income countries.^21^ The point remains that the cooccurrence of smoking and socio-economic status is a challenge for conventional analyses.

Smoking is also more prevalent amongst groups defined by some health statuses. For example, smoking is more common amongst individuals with depression and schizophrenia.^22–25^ These associations may affect all or some of smoking initiation, smoking intensity, and smoking cessation. Smoking also influences disease incidence (such as lung cancer) which in turn may prompt cessation. Smoking may reflect elements of self-medication^26^ or a desire to control one’s weight.^27^ Smoking may therefore be both a cause and consequence of health status and other circumstances.^28,29^ Measured associations of smoking with health care cost may also partly reflect wider attitudes to risk tolerance, including impulsivity and behavioral disinhibition.

Analysis of the effects of smoking may also be complicated by measurement error, including from inaccurate recollection of smoking history. Moreover, self-reported smoking patterns (“two packs a day for 15 years”) may not fully capture the consequences of smoking on health. Instead, the cumulative physiological insult of lifetime smoking on respiratory and other functions may be a better measure for this exposure.

We used Mendelian Randomization to address these challenges. Subject to the assumptions of instrumental variable analysis, causal effects of smoking on health care cost can be robustly identified by perturbations to genetic variants associated with the liability to smoke cigarettes. Since this genetic variation is fixed at conception, it cannot be affected by reverse causation. By virtue of the quasi-random allocation of genetic variation at conception, it will be independent of many confounding variables that might otherwise affect the association between smoking and health care costs. We attempted to account for measurement error by using a recently developed composite smoking index that reflects the impact of both smoking duration (encompassing initiation and cessation) and smoking intensity on mortality and health.

However, Mendelian Randomization does not avoid all challenges connected with more conventional methods. As in conventional analysis, it may also be subject to selection bias of various types, including with respect to who self-selects into volunteer cohorts (such as the UK Biobank cohort which we use as the source of outcome data), and with respect to which individuals live long enough to be represented in these cohorts despite exposure to smoking. Mendelian Randomization also bears its own set of assumptions, some of which are untestable. The results of these first Mendelian Randomization analyses will have particular value when triangulated alongside other analytic methods that rely on different sets of assumptions.

## Methods

Many introductions to Mendelian Randomization are available (eg^30,31^). Briefly, individuals have two alleles (specific genetic code at a particular location or locus in the genome) at each location, one inherited from each parent according to Mendel’s first law (the random segregation of alleles) and second law (independent assortment of alleles for different traits) of inheritance. Single nucleotide polymorphisms (SNPs) are examples of genetic variation subject to Mendel’s first and second laws. SNPs are single changes to the nucleotides that make up the genomic code. A variety of different SNPs have been found to robustly associate with various smoking phenotypes—see, for example^28,32^

### Conventional Multivariable Analysis

We implemented conventional multivariable linear regression as well as Mendelian Randomization. The conventional linear regression models related health care cost to both smoking status phenotypes, controlling in each case for age, sex, and Biobank recruitment center.

### Instrumental Variable Analysis

The principal instrumental variable models were calculated using versions of the Wald ratio. In Mendelian Randomization, the Wald ratio is calculated as the ratio of the association between the SNP(s) and outcome to the association between the SNPs and the exposure. This ratio is equivalent to the two-stage least squares (2SLS) model when a single instrument is used. The first stage of a 2SLS regresses an exposure variable on the instrument variables. The predicted values of the smoking exposures from this regression are then used in the second stage regression with costs as the outcome. The *F*-statistic from the first stage regression tests the joint significance of all instruments and is an indicator of instrument strength. Weak instruments will lead to estimates biased in the direction of the conventional multivariable estimate.

For the 2SLS models, we developed polygenic risk scores (PRSs), also known as genetic risk scores or allele scores. Each PRS was calculated as the weighted sum of the effect alleles for all SNPs. Each SNP was weighted by the regression coefficient from the respective GWAS in which the SNP was identified. These estimates were adjusted with age, sex, and the first 40 genetic principal components.

The Mendelian randomization models we estimate can be interpreted as additive structural mean models.^33^ For the composite smoking index, these estimates represent the mean change in the cost outcome caused by a one-unit genetically influenced increase in the value of the index averaged overall individuals in the sample. For the initiation phenotype, these represent the change in costs for a change in genetic liability to initiate smoking.

The interpretation of the 2SLS effect estimates is that of a unit change in genetic liability to the exposure variable. For the initiation exposure, this reflects a change in genetic liability in case status; that is, the causal effect estimate represents the average costs per person, per year of becoming a smoker. We estimated 2SLS models for two smoking phenotypes: smoking initiation and the composite smoking index.

### Smoking Phenotypes Used as Exposure Variables

Both the conventional and the Mendelian Randomization analyses used the same two smoking phenotypes as exposure variables. The first of these phenotypes is a compositive index of lifetime smoking behaviors. The phenotype is an initiation phenotype.

The composite smoking index was created by Wootton and colleagues^34^ to reflect smoking initiation, duration of smoking, heaviness of smoking, and smoking cessation (if any). These different measures were aggregated into a composite smoking index with a half-life constant to reflect the exponential declining impact of smoking on health at a given time. The half-life was obtained from a simulation of the effect of smoking on lung cancer and on all-cause mortality in the UK Biobank. The composite smoking index has been shown to have an inverse association with lung function, and a positive association with incident chronic obstructive pulmonary disease.^35^ It has also been used in the study of other exposures and outcomes, including in relation to schizophrenia and depression,^34^ bipolar disorder,^36^ inflammatory bowel syndrome,^37^ and breast and colorectal cancers.^38^

The smoking initiation phenotype measure captures whether cohort participants ever smoked, without further distinction according to duration or intensity. A binary variable indicating smoking initiation (“ever” smoked vs. “never” smoked) was created from the composite smoking index, with non-zero values of the composite smoking index indicating an individual had initiated smoking. Inference from all 2SLS models (and those of the sensitivity analyses we describe below) is conditional on the ages (mean 57 years at baseline, range from 39 to 72) of the individuals represented in our cohort.

Conventional multivariable regression models estimate the impact of a unit change in either phenotype, which in the case of the initiation phenotype reflects the effect of ever versus never smoking, and in the case of the composite index effect estimates refer to a unit increase in the value of the index. The Mendelian Randomization effect estimates have a similar interpretation, except they refer to the effect of genetic liability either to ever smoke (for the initiation phenotype) or to a unit increase in the composite smoking index.

### Sensitivity Analysis

An important potential violation of the requirements for valid instrumental variable analysis in Mendelian Randomization is horizontal pleiotropy, in which a variant is associated with the outcome other than via the exposure of interest. The presence of pleiotropy may be indicated by the presence of heterogeneity in effect estimates across SNPs. This may be tested by comparing Cochran’s *Q* statistic $Q=\underset{j=1}{\overset{J}{\sum }}\frac{1}{\sigma_{Y_{j}}^{2}}{\left({\hat{\beta }}_{j}-{\hat{\beta }}_{IVW}\right)}^{2}$ to the critical values of a chi-squared distribution. Here, *J* indexes the number of SNPs, ${\hat{\beta }}_{j}$ is the effect estimate for SNP *j*, ${\hat{\beta }}_{IVW}$ is the overall inverse variance (IVW) weighted effect calculated for *J* SNPs, and the variance of the SNP-outcome association is denoted by $\sigma_{Y_{j}}^{2}$.

We implemented a random-effects inverse-variance-weighted (IVW) estimator by calculating the Wald ratio for each SNP separately, and then combining these SNPs using weights determined by the precision of the association between each SNP and health care costs. This estimator assumes that no pleiotropy in violation of the exclusion restriction is present, or that any such pleiotropy has a net zero effect on point estimates. We implemented three other estimators that relax this assumption.

The Mendelian Randomization Egger estimator includes and conditions on an intercept, which may be interpreted as the average pleiotropic effect of all included SNPs. Horizontal pleiotropy is indicated if this intercept is statistically different from zero. This estimator is consistent even if all SNPs violate the exclusion restriction, provided further assumptions on the relationship between instrument strength and any direct pleiotropic effect of SNPs are met.^39^ These further assumptions include^40^ no direct effect of pleiotropic SNPs on the outcome, and no influence of SNPs on omitted variables in the exposure-outcome association.

The median estimate of the ordered Wald estimates will be consistent if at least 50% of SNPs are valid instrumental variables.^41^ We implemented a precision-weighted version of a median estimator in which outlying variants (measured by their contribution to the Cochran *Q* heterogeneity statistic) are penalized by down-weighting their contribution to the overall effect estimate. This weighted estimator will be consistent if at least 50% of the weight comes from valid instruments, and no single instrument is responsible for more than 50% of the weight. The mode estimator^42^ is given by the mode of the Wald ratio estimates. This estimator is consistent even if more than 50% of the SNPs are invalid instrumental variables, provided that the largest homogenous cluster of SNPs are valid instrumental variables.

We also assessed whether genetic variants associated with smoking and smoking heaviness affected costs in “ever smoker” compared to “never-smokers”—if these variants influence costs only via smoking heaviness, their effect should only be apparent in ever-smokers.^43^ We assessed this association by interacting in each of the split GWAS samples with the smoking initiation phenotype with variants associated with smoking located closest to the CHRNA5 locus. Evidence of an interaction in smokers but not non-smokers would constitute some evidence—albeit not definitive evidence—in support of the exclusion restriction for the initiation phenotype.

Smoking SNPs identified in GWASs may also reflect genetic liability to risk tolerance. Risk tolerance may therefore influence smoking behavior, as well as other risk-taking behaviors that themselves cause health care costs. We therefore implemented multivariable Mendelian Randomization, in which the casual effect of both smoking behavior and risk tolerance were jointly estimated in a single instrumental variable model. Multivariable Mendelian Randomization allows for the direct effect (not via the other exposure) of each exposure to be determined. We implemented separate multivariable Mendelian Randomization models for smoking initiation and risk tolerance and for the composite smoking index and risk tolerance. We used the methods of Sanderson and colleagues^44^ to implement our models.

We applied Steiger filtering^45^ to each smoking exposure as a simple test of the assumption that instruments affect the outcome via the influence of these instruments on the exposure. This test calculates the variance explained by the SNPs, and tests if the variance explained by the instrument in the outcome is less than the variance explained in the exposure. If so, this provides some evidence that the SNPs influence the exposure via the outcome, and not vice versa.

## Data

We used the UK Biobank study as our primary source of data.^46^ This is a population-based cohort of some 500 000 individuals recruited between 2006 and 2010. All adults aged 40–69 living in defined catchment areas were invited to participate,^46^ with a final response rate of 5.45%. Participants provided a wide variety of personal and phenotypic data at baseline recruitment, and most consented to genotyping.

Mendelian Randomization studies may be biased if the same sample used to select SNPs is also used as the sample in which those SNPs are analyzed as instrumental variables. Sample overlap will tend to bias associations towards the observational exposure-outcome association. We therefore randomly split our available Biobank sample into two non-overlapping samples. On one of these sets, we conducted de novo GWASs to identify SNPs, which we then analyzed on the other set of data. We then performed a fixed-effect meta-analysis to obtain a single summary measure of effect (and the associated confidence intervals) across both samples. We used a set of standard in-house^47,48^ quality control procedures for conducting the de novo UK Biobank GWASs. We used a clumping threshold of *R*^2^ < 0·001 to account for potential linkage disequilibrium between SNPs.

Genetic data on the composite smoking index (and the related binary smoking initiation variable) was obtained from two de novo GWASs of using the composite smoking index undertaken on 318 067 individuals in the UK Biobank. We also conducted a de novo GWAS for a measure of risk tolerance to use in multivariable Mendelian Randomization (*N* = 274 450). This was constructed using responses to the UK Biobank questionnaires. A score was created from response to questions relating to days per week of moderate and vigorous physical activity, hours of TV viewing, breaking of motorway speed limits, illicit drug use, alcohol consumption, self-harm, and sexual activity. Details concerning the construction of this variable are given in Supplementary Material.

Genetic and other data from the UK Biobank were linked to records of inpatient hospital care from which costs per person, per year of follow-up in the UK Biobank were calculated. A patient undergoing an inpatient hospital visit will occupy a bed but does not necessarily stay overnight. The process by which episodes of care were coded to reflect costs are described elsewhere.^49,50^ Briefly, episodes of inpatient hospital care were coded to create hospital resource groups (HRGs), which denote episodes of care with similar diagnoses, operations, and procedures. These HRGs were then cross-referenced to unit costs of care to create a per-person, per-year overall figure for inpatient hospital costs. These electronic health records of inpatient care episodes are comprehensive, reflecting all care provided to public and private patients in National Health Service (NHS) hospitals for the full period of follow-up, although it does not reflect the very small proportion of care that may have been received by private patients in private hospitals.

All analyses were performed in *R* version 4.02 and Stata version 16.1. Analysis code is available at www.github.com/pdixon-econ/MR_smoking_costs.

## Results

Up to 300,045 individuals were analyzed, of whom 54% were female (n = 161,022). Mean age at recruitment was 57 years (standard deviation = 8.0 years). Mean costs per year were £478 and median costs £87. Some 45% of the sample had zero inpatient NHS costs. There were 87 651 individuals in this sample who had ever smoked. The range of the composite smoking index is from 0 to 4.0, with a score of 0 indicating a never-smoker. The mean value of this index amongst smokers was 1.14 (standard deviation: 0.78).

Conventional multivariable models, estimated using linear regression and adjusted for age and sex but without any genetic information, are summarized in Table 1.

**Table 1.** Multivariable Estimates of the Association Between Smoking Exposures (Smoking Initiation and Lifetime Smoking), and Annual Inpatient Hospital Costs

	*N*	Effect estimate	95% confidence interval
Phenotype			
Smoking initiation	299 714	£183	£171 to £195
Composite smoking index	299 714	£112	£106 to £119
Composite smoking index amongst smokers	87 563	£125	£114 to £137

Notes: These models adjusted for age, sex, the Townsend deprivation index, and recruitment center. The effect estimate for the smoking initiation phenotype reflects the impact on annual inpatient hospital costs of ever versus never smoking. The composite smoking index phenotype effect estimate reflects the impact of an additional unit on the composite smoking index on annual inpatient hospital costs.

The effect estimate (£183) on the smoking initiation phenotype indicates the observational association between becoming a smoker compared to never smoking on per-person, per-year inpatient hospital costs. Likewise, a unit change in the composite smoking index in the intensity, duration, and cessation (if any) is associated with £125 increase in annual per-person inpatient costs. Given that median per-person costs in this sample are £87 per year, both smoking exposures are associated with a substantial increase in annual costs. These conventional multivariable models will be confounded by any variables other than age, sex, and the UK Biobank recruitment center that jointly influence smoking and health care costs.

Table 2 details information relating to the polygenic instrumental variable models.

**Table 2.** Cases, Number of SNPs, and Strength of Instruments Used in Mendelian Randomization Analysis for all Smoking Exposures

	*N*	No. of SNPs	% of variance explained by polygenic risk score	*F*-statistic from first stage of 2SLS polygenic risk score 2SLS model
Phenotype				
Smoking initiation sample 1	149 995	10	0.19%	223
Smoking initiation sample 2	150 050	11	0.25%	293
Composite smoking index sample 1	149 995	17	0.40%	584
Composite smoking index sample 2	150 050	15	0.44%	620

Notes: Percentage of variance obtained from pseudo *R*^2^ from a logistic regression of initiation status on the PRS for initiation, and from *R*^2^ obtained from linear regression of the composite smoking index on the PRS for this exposure.

The number of SNPs refers to genome-wide significant hits in each split sample of the UK Biobank cohort. The proportion of variance explained by the polygenic risks scores is modest, being less than 1% in all cases. However, the first stage F-statistic from the 2SLS indicates that these are strong instruments.

### Instrumental Variable Results

Table 3 summarizes the causal effect estimates and associated 95% confidence intervals representing the effect of genetic liability to each smoking phenotype, estimated using polygenic risk score 2SLS models.

**Table 3.** 2SLS Allele Score Mendelian Randomization Estimates for all Smoking Exposures

	Beta	95% confidence interval
Phenotype		
Smoking initiation	£477	£187 to £766
Composite smoking index	£204	£105 to £303

Notes: Estimates and confidence intervals from fixed-effects meta-analysis over each split sample. The effect estimate for the smoking initiation phenotype reflects the impact on annual inpatient hospital costs of ever versus never smoking. The composite smoking index phenotype effect estimate reflects the impact of an additional unit on the composite smoking index on annual inpatient hospital costs.

The betas in Table 3 reflect inpatient hospital costs per person per year of a (genetically influenced) change in liability to smoking initiation and to the composite smoking index. Note that these represent the effect of genetic liability on each smoking phenotype, and are not directly comparable to the corresponding conventional multivariable estimates.^51,52^ Note also that the wide confidence intervals are a consequence of the modest proportion of variance that each polygenic risk scores explains in the respective phenotypes. The confidence intervals for the binary initiation phenotype overlap with the composite smoking index phenotype, and the phenotypes are on different scales, which therefore does not necessarily suggest that initiation is more “expensive” than a unit change in the composite exposure phenotype. Nevertheless, the effect estimates are consistent with the substantial effects of genetic liability to each smoking phenotype on health care costs. Note also that, as with the observational analyses reported in Table 2, these may be affected by the non-survival of smokers to middle- and early-old age, since only smokers who survived to this point could have been recruited into the UK Biobank.

### Sensitivity Analysis

Inspection of Cochran’s *Q* revealed little evidence of heterogeneity (Table 4), with the exception of the composite smoking index in the first split sample. This may indicate a violation of instrumental variable assumptions for this exposure, and caution is required in interpreting results from these models. We report the findings of the various pleiotropy-robust estimators in Supplementary Material. The point estimates from these models are associated with considerable uncertainty, and no clear pattern of association between the smoking phenotypes and health care cost is apparent. Again, this suggests caution is merited in interpreting the results of, in particular, the composite smoking index results.

**Table 4.** Cochran’s *Q* Statistic and Heterogeneity by Phenotype for all Smoking Exposures

	No. of SNPs	*Q* statistic	*Q p*-value
Phenotype			
Smoking initiation sample 1	10	10.6	.31
Smoking initiation sample 2	11	16.2	.06
Composite smoking index sample 1	17	34.1	<.01
Composite smoking index sample 2	15	16.8	.21

Note: There were insufficient SNPs to calculate the *Q* statistic for the risk tolerance exposure.

Supplementary Material also contains the results of Steiger filtering tests, which indicated that the direction of causality was more likely to be from each smoking exposure to costs rather than vice versa. Interactions between initiation and variants located near the *CHRNA5* locus were consistent with the null, which is further tentative evidence that the exclusion restriction was not violated for the initiation phenotype.

For multivariable Mendelian Randomization, SNPs must be strongly associated with each exposure, conditional on the other included exposures. Sanderson and colleagues^44^ recommend that each exposure included have an *F*-statistic greater than 10. In the event, the conditional strengths of the SNPs for risk tolerance (but not the two smoking exposures) were too weak to make reliable inferences. The results of these analyses are reported in Supplementary Material.

## Discussion

We analyzed the impact of smoking exposures on health care costs using genetic instrumental variables. There is little to no causal evidence from other quasi-experimental study designs regarding these associations.^53^ The systematic review of Makate et al^54^ indicates that most studies examining these associations have generally relied on controlling for observable characteristics in cross-sectional study designs or longitudinal study designs, often using aggregate rather than individual level data. These studies are likely to be confounded by omitted variables that jointly influence both smoking status and health care cost.

For example, de Boer et al^55^ use aggregate data to note that neighborhoods with lower rates of smoking were associated with lower health care costs. However, neighborhoods with higher rates of smoking are likely to differ because of unobserved and unmeasured covariates that would likely increase average health care costs compared to “healthier” neighborhoods even in the absence of differential rates of smoking. Xu et al^56,57^ include controls based on body mass index in their models of the association between smoking status and health care costs. Since body mass index influences and is influenced by smoking status, the causal interpretation of results from this type of study design is challenged if BMI is a collider or “bad control” in this context.

Conventional multivariable estimates in simple linear models indicated a substantial association between both smoking initiation and the composite smoking index on annual per-patient inpatient costs in the UK Biobank cohort, as did the 2SLS Mendelian Randomization models. Mendelian Randomization analyses were estimated with considerable uncertainty. There was some evidence that the composite smoking index models may have violated the exclusion restriction assumption. Results from the pleiotropy-robust estimators were associated with uncertainty and did not indicate a clear direction or magnitude of causal effects for either smoking exposure. This uncertainty should be borne in mind when interpreting all of the Mendelian Randomization results.

Comparisons between conventional and Mendelian Randomization estimates are not necessarily straightforward. The conventional estimates amount to a comparison of costs between groups defined by particular forms of smoking behavior. The Mendelian Randomization involves a similar comparison but in which the distinction between groups is defined by genetic liability to smoking. Groups defined on these distinct bases may not be comparable. Neither type of method, whether relying on conventional methods or Mendelian Randomization, necessarily corresponds with the effect estimates that would be obtained from a hypothetical experiment to compel people to smoke (or to compel smoking cessation) and in which health care costs are observed over a period of time. Mendelian Randomization and conventional estimates therefore may not comply with the stable unit treatment value assumption (SUTVA) of causal inference.^58^

Our Mendelian Randomization effect estimates relate to the cumulative (from conception) impact of a genetic liability to the smoking phenotypes we study, and cannot necessarily be used to make inferences about the effects of smoking at particular phases of life or over the entire life course, or on other smoking phenotypes. The Mendelian Randomization results provide evidence on the causal impact of smoking at middle- and early-old age amongst individuals in the UK Biobank cohort, and cannot necessarily be used to infer the lifetime impact of smoking in other groups. The principal use of our estimates is likely to be as inputs into decision analytic models, and as sources of evidence that may be used to triangulate^59^ other findings on the impacts of smoking.

The results (of both the conventional and Mendelian Randomization analyses) are also specific to the context of health care and smoking behaviors in the UK Biobank, and may not have direct relevance when translated to other national contexts and to other health care systems. We were also limited to studying individuals of European (White British) ancestry. The relationships we estimate on this ethnicity may not reflect associations between smoking and costs in other ancestral groups.

Under the assumption of monotonicity—that the effect of the instrumental variables on each respective exposure is in the same direction for all subjects—these estimates are local average treatment effect estimates that capture lifelong genetic liability to smoke. In the present study, this assumption means for those individuals whose smoking differs by levels of the respective instruments, then the association change in smoking behavior is in the same direction for all individuals. This assumption may be reasonable although we do not formally test it.

Our analysis was restricted to inpatient costs only. This means other costs causally associated with smoking elsewhere in the health system—for example in outpatient clinics and in primary care—do not influence our estimates. Our results therefore understate the impact of smoking on total health care costs, since these other forms of care are much more likely to be complements than substitutes for the inpatient care costs that we study.

There was some evidence of heterogeneity associated with pleiotropy in our analysis of the composite smoking index. If risk attitudes (or other variables) confound the association between smoking and health care costs, then interventions that address smoking may not necessarily influence health care costs. Moreover, confounding the effects of smoking on health care cost with those of attitudes toward risk may lead to biased estimates of the external effects of smoking. There was no attenuation of the effects of smoking on health care cost when including instruments for risk tolerance, although this finding was not robust to weak instrument bias. We cannot rule out bias arising from associations between risk attitudes and smoking, including via correlated pleiotropy that could be due to a heritable factor common to both smoking and risk attitudes.

The UK Biobank cohort is a self-selected sample of individuals who are healthier, wealthier, and more educated than the wider UK population of adults. Smokers were less likely to participate than non-smokers,^60,61^ and lung cancer incidence in the cohort is lower than for the population from which the sample was drawn.^62^ These effects may lead to sample selection bias^63,64^ for both conventional and Mendelian Randomization estimates.

Van Alten et al^65^ created inverse probability weights based on census micro data to account for volunteer bias in the UK Biobank. Individuals would be more likely to report smoking (and other addictive behaviors) when the cohort is reweighted in this fashion. This could indicate that our results could underestimate the impact of smoking in the general population. Schoeler et al^64^ also evaluated the impact of non-random participation in the UK Biobank, including with respect to smoking status (never/ever/current). They found that SNP effects for smoking status were likely to be overestimated in GWASs that did not weigh for participation. There was no evidence of bias from changes in the direction of Mendelian Randomization estimates although some 6% of these Mendelian Randomization estimates were either overestimated (2%) or underestimated (4%). Just two of 234 associations (including the effect of a smoking status exposure on fruit consumption) indicated significant (false discovery-corrected *p*-values <.05) differential effects between the conventional and inverse-variance-weighted estimates.

The overall impact on the Mendelian Randomization estimates from using SNPs unweighted for participation is therefore somewhat uncertain, but we cannot rule out possible biases in our analysis. Simulation studies^66^ suggest that biases other than those attributable to selection, such as violations of the exclusion restriction caused by pleiotropy, may have a greater impact on effect estimates. Selection bias can also arise when the sample is chosen based on surviving to recruitment with respect to the genetically instrumented exposure and competing risks associated with the outcome. For example, smoking may influence the risk of lung cancer and heart disease, which are competing risk factors for practically every other health condition that would influence inpatient hospital costs in early- and middle-old age. If present, this type of bias may lead us to underestimate the causal effect of smoking on health care costs in later life. Schooling et al^67^ note that adjusting for common factors influencing both survival and the outcome in a multivariable Mendelian Randomization analysis can, in some cases, help alleviate this type of selection bias. Given our binary initiation phenotype, the smaller sample size for individuals for whom the composite smoking exposure phenotype was measured, and our split sample design, we did not attempt to implement these methods, although they appear promising for quantifying this type of selection bias for future studies analyzing larger samples of smokers.

Mendelian Randomization requires that variants are conditionally independent of local environments. This assumption will be violated if there is clustering of alleles in certain environments, or amongst certain types of individual. In relation to the former, geographical stratification that is not wholly accounted for by genetic principal components is a feature of the UK Biobank^68^ and may impart bias to our results. In relation to the latter, alleles that indicate genetic liability to smoking will tend to cluster amongst individuals if assortative mating means that smokers are more likely to marry other smokers than they are to marry non-smokers.^69^

Children raised by smokers may be more likely to smoke independently of the genetic liability that a child has to smoke. Howe et al^70^ use a within-family GWAS to control for demographic and indirect genetic effects, such as the impact of non-transmitted parental alleles. These GWAS found smaller effect estimates for smoking than in GWASs of unrelated individuals. This would suggest that the effect estimates in the present study, drawn from GWASs of unrelated individuals, may be upward biased.

## Conclusions

Mendelian Randomization analysis of the causal effect of genetic liability smoking initiation and a composite smoking index in a cohort of middle-aged and early old-age individuals indicated a substantial impact of each exposure on annual inpatient hospital costs, although these associations may be inflated by indirect genetic effects and potentially also by risk attitudes and by pleiotropy. The costs we attribute to smoking were estimated with considerable uncertainty. However, they are consistent with significant externalities to smoking and suggest efforts to prevent smoking initiation and to encourage cessation could well be cost-effective.

## Supplementary material

Supplementary material is available at *Nicotine and Tobacco Research* online.

ntae089_suppl_Supplementary_Materials

## Data Availability

The UK Biobank data may be made available to researchers following application to its data access committee. Analysis code used in this paper is available at www.github.com/pdixon-econ/MR_smoking_costs.
