# Supplementary material for: Mendelian Randomization Analysis of the Causal Effect of Cigarette Smoking on Hospital Costs
Source: Nicotine Tob Res. 2024 Apr 17;26(11):1521–9. doi: 10.1093/ntr/ntae089 (PMC11494471; doi:10.1093/ntr/ntae089)
Supplement: ntae089_suppl_Supplementary_Materials [file ntae089_suppl_supplementary_materials.docx]

# Creation of the risk tolerance phenotype

This risk phenotype was created as follows.

- Exercise: Risky behaviour was coded if <5 days per week of moderate or vigorous physical activity
- TV viewing: code >=3 hours per day coded as risky behaviour
- Driving: Ever breaking the motorway speed limit was coded as risky behaviour
- Drug use: Ever using illicit drugs was coded as risky behaviour
- Alcohol consumption: This was coded as risky behaviour if an individual reported drinking daily or almost daily

Cannabis use: Ever using cannabis was coded as risky behaviour

- Self-harm: Ever engaging in self-harming behaviour was coded as risky behaviour
- Age at first sexual intercourse: Age <16 years at first sexual intercourse was coded as risky behaviour.

Two versions of this score were created. An overall risk score was created by the sum of all responses to these questions indicating a “risky” behaviour, provided that no more than three responses to any of these questions were missing. This is the score reported in the main paper text and refers to the entire sample of individuals (n=274,450 amongst individuals of White British ancestry studied in the main inferential analysis).

The cannabis/drug use/self-harm questions were only asked at online follow up. The second score (approximately n=105,000 were eligible for inclusion in each GWAS) restricted analysis only to those reporting baseline and these online follow-up responses, and with no more than two other categories of risky behaviour missing. For the restricted score, just one SNP was identified in the first split sample, and one for the second split sample, compared to three and two respectively for the larger set. Analysis was re-run on the restricted score – results were similar to those obtained from the unrestricted score, but were even more imprecise than the unrestricted score.

# Pleiotropy-robust sensitivity analysis

This section presents the results of the two-sample summary Mendelian Randomization sensitivity analyses for each smoking exposure in each split sample. Note that the effect estimates are on the scale of the respective genome wide association studies. The composite smoking index was analyzed in its GWAS as a continuous variable on a linear scale. Logistic regression was used to analyze the binary outcome of smoking initiation in each GWAS. The effect estimates for initiation in the tables below therefore correspond to the change in costs per unit change in the log-odds of initiating smoking. A unit change is therefore exp(1), which is 2.72-fold change on the multiplicative scale in the odds of initiating smoking.

For reference, we include tables with the conventional multivariable (Table A1) and 2SLS Mendelian Randomization (A2).

**Table A1 Smoking initiation: Results of conventional multivariable analysis (reproduced from the main paper for reference)**

|  | **N** | **Effect estimate** | **95% confidence interval** |
| --- | --- | --- | --- |
| **Phenotype** | | | |
| Smoking initiation | 299,714 | £183 | £171 to £195 |
| Composite smoking index | 299,714 | £112 | £106 to £119 |
| Composite smoking index amongst smokers | 87,563 | £125 | £114 to £137 |

**Table A2 Smoking initiation: Results of 2SLS Mendelian Randomization analysis (reproduced from the main paper for reference)**

**Beta 95% confidence interval**

| **Phenotype** |  |  |
| --- | --- | --- |
| Smoking initiation | £477 | £187 to £766 |
| Composite smoking index | £204 | £105 to £303 |

**Table A3 Smoking initiation: Results of summary Mendelian Randomization sensitivity analysis**

|  | **Estimate** | **Standard error** | **P-value** |
| --- | --- | --- | --- |
| **Smoking initiation sample 1** | | | |
| Inverse variance weighted | -£248 | £138 | 0.07 |
| MR Egger | -£497 | £1101 | 0.66 |
| Penalized weighted median | -£274 | £175 | 0.12 |
| Weighted mode | -£268 | £280 | 0.36 |
| **Smoking initiation sample 2** | | | |
| Inverse variance weighted | £178 | £175 | 0.31 |
| MR Egger | £641 | £987 | 0.53 |
| Penalized weighted median | £311 | £193 | 0.11 |
| Weighted mode | £468 | £295 | 0.15 |

**Table A4 Composite smoking index: Results of summary Mendelian Randomization sensitivity analysis**

|  | **Estimate** | **Standard error** | **P-value** |
| --- | --- | --- | --- |
| **Composite smoking index sample 1** | | | |
| Inverse variance weighted | £11 | £101 | 0.91 |
| MR Egger | -£313 | £467 | 0.51 |
| Penalized weighted median | -£120 | £108 | 0.27 |
| Weighted mode | -£166 | £199 | 0.42 |
| **Composite smoking index sample 2** | | | |
| Inverse variance weighted | £68 | £83 | 0.41 |
| MR Egger | -£174 | £264 | 0.52 |
| Penalized weighted median | -£80 | £143 | 0.44 |
| Weighted mode | -£144 | £177 | 0.43 |

Results for all estimators were associated with considerable uncertainty and are consistent with the null for both phenotypes for all estimates in each sample. Point estimates indicate both positive and negative impacts of smoking on healthcare costs, but these must be interpreted in the context of very broad confidence intervals.

# Results of Steiger filtering

**Table A3 Steiger filtering results for smoking phenotypes**

|  | **SNP R^2^ exposure** | **SNP R^2^ outcome** | **P-value** |
| --- | --- | --- | --- |
| **Smoking initiation** | | | |
| Sample 1 | 0.003 | <0.001 | <0.001 |
| Sample 2 | 0.003 | <0.001 | <0.001 |
| **Composite smoking index** | | | |
| Sample 1 | 0.004 | <0.001 | <0.001 |
| Sample 2 | 0.004 | <0.001 | <0.001 |

# Results of smoking initiation interaction test

In each split GWAS sample, we identified SNPs on chromosome 15 and close to the CHRNA5 locus for which their strongest associations in previous studies reported in publicly available databases (including dbSNP and MR Base) were with smoking phenotypes. In sample 1, we used the rs7173514 SNP and in sample 2 rs28681284. For each sample, we estimated linear regressions of the cost outcome on each respective SNP, the smoking phenotype, the interaction of the SNP with the smoking initiation phenotype, and controls for age, sex, UK Biobank assessment center, and the first forty principal components. In each case, the interaction was consistent with the null. For sample 1, the effect of the interaction was -£11.92 (95% confidence interval -£34.61 to £10.77), and for sample 2 £22.41 (95% confidence interval: -£1.32 to £46.14).

# Multivariable Mendelian Randomization analysis

## A directed acyclic graph for multivariable Mendelian Randomization

A representation of the multivariable Mendelian Randomization model (based on [1]) for two exposures (X_1_ to X_2_) is shown in Figure A4.

**Figure A4 Multivariable Mendelian Randomization**

Here, G is a set of SNPs that influences both exposures. In a conventional or univariable Mendelian Randomization analysis, the causal effect estimate reflects the “total” effect of the exposure on an outcome. In a multivariable Mendelian Randomization, the effect estimate for each exposure represents a “direct” effect (respectively β_1_ and β_2_) on the outcome. The sum of the direct effects may not be the same as the total effect, since this depends on the nature of the association between the exposures [1]. This is indicated by the dashed line from X_1_ to X_2._

The model estimated is of the form:

$Y=\alpha_{0}+\beta_{1}X_{1}+\beta_{2}X_{2}+\upsilon_{y}$

Here, Y is the outcome, $\alpha_{0}$ is an intercept term, X1 and X2 are exposures that in our analysis represent smoking and risk tolerance. This equation is estimated using two-stage least squares regression, where the first stage regression predicts both exposures from the full set of SNPs that relate to each exposure.

## Results of multivariable Mendelian Randomization

Three SNPs were genome-wide significant in the first split sample (N=129,864) for this phenotype, and just two SNPs in the second split sample (N=129,660). The polygenic risk scores created from these SNPs explained 0.10% and 0.05% in the first and second samples respectively. These SNPs were associated with first stage 2SLS F-statistics of 39 and 28.

The F-statistic for risk tolerance in a multivariable Mendelian Randomization with the compositive index was 20.2 in the first sample, and 10.7 in the second, compared to 15.0 and 10.3 for the smoking initiation exposure in each respective sample. The F-statistic for risk tolerance in a multivariable Mendelian Randomization with the continuous lifetime smoking index was 7.1 in the first sample and 2.8 in the second, compared to 6.1 and 2.7 for smoking initiation in each respective sample.

Table A4 summarizes the results of the multivariable Mendelian Randomization analysis for each smoking exposure and for each sample. We emphasize that the results are not robust to possible weak instrument bias (as noted in the main text) and are reported here for completeness.

**Table A4 Results of multivariable Mendelian Randomization**

|  | **Estimate** | **Standard error** | **P-value** |
| --- | --- | --- | --- |
| **Initiation** |  |  |  |
| Smoking initiation sample 1 | £272 | £126 | 0.04 |
| Risk tolerance sample 1 | -£118 | £93 | 0.21 |
| Smoking initiation sample 2 | £381 | £95 | <0.01 |
| Risk tolerance sample 2 | -£68 | £112 | 0.54 |
| **Composite smoking index** | | | |
| Composite smoking index sample 1 | £198 | £76 | 0.02 |
| Risk tolerance sample 1 | -£118 | £86 | 0.18 |
| Composite smoking index sample 2 | £224 | £61 | <0.01 |
| Risk tolerance sample 2 | -£29 | £112 | 0.80 |

# Supplementary material references

1. Sanderson E, Davey Smith G, Windmeijer F, Bowden J. An examination of multivariable Mendelian randomization in the single-sample and two-sample summary data settings. Int J Epidemiol. 2019;48(3):713-27.
